# Development and Validation of a Predictive Model for Early Refractoriness of Transarterial Chemoembolization in Patients With Hepatocellular Carcinoma

**DOI:** 10.3389/fmolb.2021.633590

**Published:** 2021-03-18

**Authors:** Tian-Cheng Wang, Tian-Zhi An, Jun-Xiang Li, Zi-Shu Zhang, Yu-Dong Xiao

**Affiliations:** ^1^ Department of Radiology, Secong Xiangya Hospital, Central South University, Changsha, China; ^2^ Department of Interventional Radiology, The Affiliated Hospital of Guizhou Medical University, Guiyang, China; ^3^ Department of Interventional Radiology, Guizhou Medical University Affiliated Cancer Hospital, Guiyang, China

**Keywords:** hepatocellular carcinoma, treatment failure, predictive, logistic regression, transarterial chemoembolization

## Abstract

**Objectives:** To develop and validate a predictive model for early refractoriness of transarterial chemoembolization (TACE) in patients with hepatocellular carcinoma (HCC).

**Methods:** In this multicenter retrospective study, a total of 204 consecutive patients who initially underwent TACE were included. Early TACE refractoriness was defined as patients presented with TACE refractoriness after initial two consecutive TACE procedures. Of all patients, 147 patients (approximately 70%) were assigned to a training set, and the remaining 57 patients (approximately 30%) were assigned to a validation set. Predictive model was established using forward stepwise logistic regression and nomogram. Based on factors selected by logistic regression, a one-to-one propensity score matching (PSM) was conducted to compare progression-free survival (PFS) between patients who were present or absent of early TACE refractoriness. PFS curve was estimated by Kaplan-Meier method and compared by log-rank test.

**Results:** Logistic regression revealed that bilobar tumor distribution (*p* = 0.002), more than three tumors (*p* = 0.005) and beyond up-to-seven criteria (*p* = 0.001) were significantly related to early TACE refractoriness. The discriminative abilities, as determined by the area under the receiver operating characteristic (ROC) curve, were 0.788 in the training cohort and 0.706 in the validation cohort. After PSM, the result showed that patients who were absent of early TACE refractoriness had a significantly higher PFS rate than those of patients who were present (*p* < 0.001).

**Conclusion:** This study presents a predictive model with moderate accuracy to identify patients with high risk of early TACE refractoriness, and patients with early TACE refractoriness may have a poor prognosis.

## Introduction

Hepatocellular carcinoma (HCC) is a common alimentary malignancy worldwide (Long et al., 2020; Peisen et al., 2020). For patients with HCC, ablative therapy, surgical resection, and liver transplantation are the potentially curative treatments. However, most patients are diagnosed with an advanced stage of disease, and only 20–30% of patients can receive curative treatments (Arizumi et al., 2015; Eilard et al., 2019; Peng et al., 2019; Luedemann et al., 2020). Transarterial chemoembolization (TACE) is the standard therapy for intermediate-stage HCC, which is accepted by several guidelines (Piscaglia and Ogasawara, 2018; Lee et al., 2019). However, it has been reported that not all HCC patients respond to TACE because the patients selected for TACE correspond to a highly heterogeneous population, covering a wide range of tumor burdens, liver function and treatment histories (Maesaka et al., 2020; Xue et al., 2020). Furthermore, repeated TACE procedures could gradually lead to TACE refractoriness, and some patients even show TACE failure at the very beginning of their treatment (Maesaka et al., 2020). For patients with TACE refractoriness, TACE is no longer effective, and those patients are recommended to switch to a systemic therapy, as suggested by the Japan Society of Hepatology (JSH) and the Liver *Cancer* Study Group of Japan (LCSGJ) (Kudo et al., 2014). Therefore, it is of great importance to identify predictive risk factors of early TACE refractoriness so that patients with those factors might switch to systemic therapy earlier to improve their survival.

Therefore, the purpose of the present study is to develop and validate a predictive model for early TACE refractoriness in patients with HCC and compare the progression-free survival (PFS) in patients who are present or absent of early TACE refractoriness.

## Materials and Methods

### Patients

This retrospective study was approved by the institutional review boards of our hospital and in accordance with the Declaration of Helsinki. The requirement for written informed consent was waived by the institutional review boards due to the retrospective nature of the present study.

**FIGURE 1:**
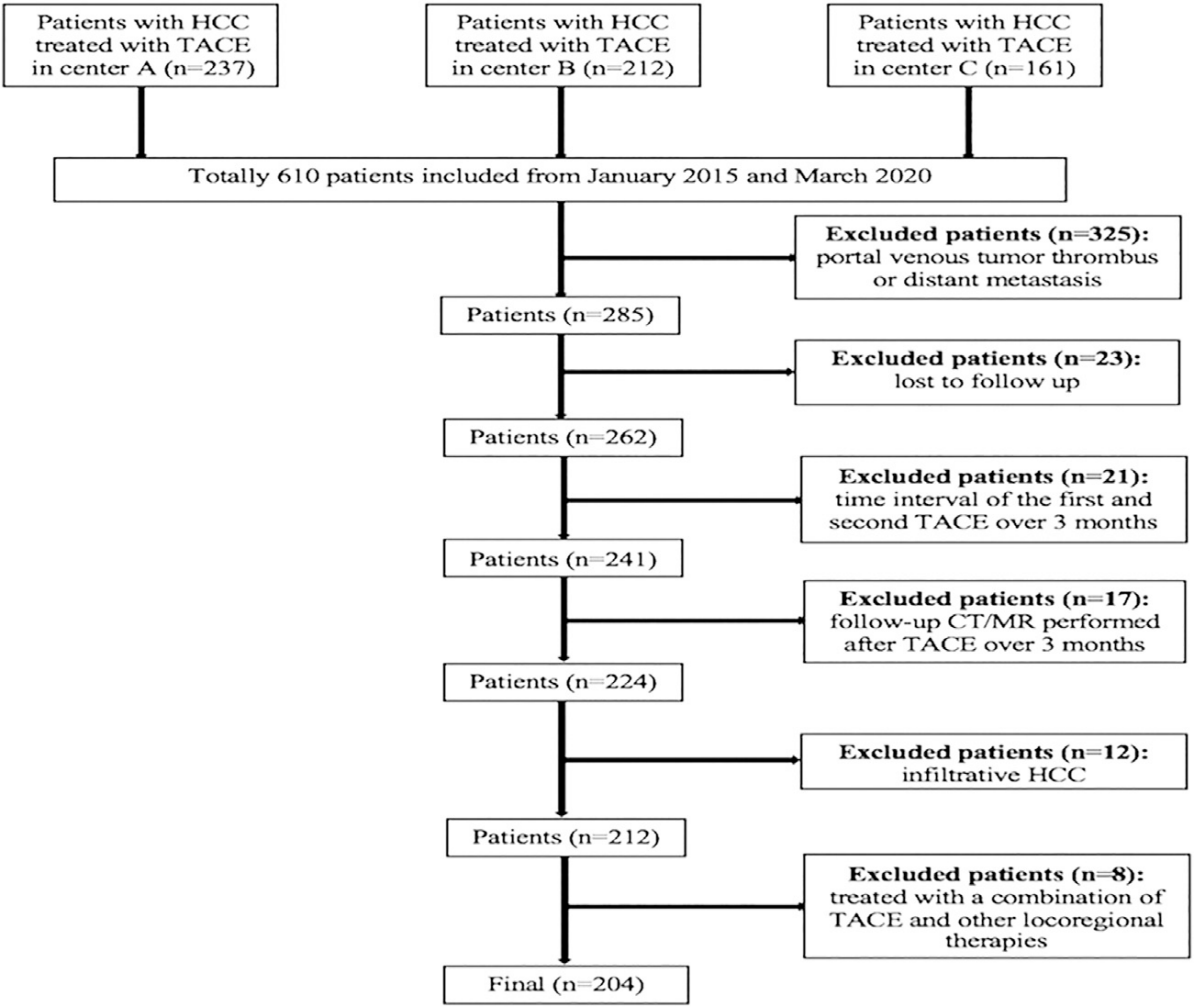
Diagram of the study population.

A total of 610 consecutive patients with unresectable HCC who initially underwent TACE at three hospitals between January 2015 and March 2020 were included. The inclusion criteria were as follows: patients had 1) Eastern Cooperative Oncology Group performance status 0; 2) compensated liver function (Child-Pugh class A or B); and 3) at least two consecutive TACE sessions, or although only one TACE session was performed, complete response (CR) was achieved after the TACE session. The exclusion criteria were as follows: patients had 1) portal venous tumor thrombus (n = 216); 2) distant metastasis (n = 109); 3) lost to follow-up (n = 23); 4) a time interval between the first and second TACE over 3 months (n = 21); 5) follow-up computed tomography (CT) or magnetic resonance (MR) imaging performed after TACE over 3 months (n = 17); 6) infiltrative HCC (n = 12); and 7) having been treated with a combination of TACE and other locoregional therapies such as ablation (n = 8). The flowchart of the study population is shown in Figure 1.

### Interventions

TACE procedures were discussed with the tumor board prior to administration for each patient. Celiac trunk and superior mesenteric arteriography, as well as indirect portography, were performed to visualize the variations in hepatic arterial anatomy and to evaluate the patency of the portal vein. Either a 2.7 French (Progreat, Terumo Medical Corporation) or a 2.2 French (Carnelian, Tokai Medical Products) coaxial microcatheter was placed into the tumor-feeding arteries with the assistance of cone beam computed tomography (CBCT) if needed. TACE was performed using either drug-eluting beads (DEB) (CalliSpheres Beads, Jiangsu Hengrui Medicine Co., Ltd.) loaded with epirubicin (Shandong New Time Pharmaceutical Co., Ltd.) or up to 20 ml emulsion of iodized oil (Lipiodol, Guerbet Asia Pacific Ltd.) mixed with epirubicin. The oil-epirubicin emulsion was created using the water-in-oil technique by mixing iodized oil with a distilled water solution containing a drug cocktail of dissolved epirubicin at a ratio of 3:1. The dosage of epirubicin in DEB-TACE ranged from 50 to 150 mg and the size of DEBs varied from 100 to 300 um and 300–500 um, while in conventional TACE the dosage of epirubicin was 50–75 mg/m^2^ body surface area. In DEB-TACE, no additional embolization was performed after injected of 1–2 g DEB, while in conventional TACE, gelfoam slurries were injected to embolize the proximal tumor feeders after the oil-epirubicin emulsion was injected. The technical endpoint of TACE was defined as the reduction in arterial inflow to the tumor and tumor devascularization. Changes in embolic agents, chemotherapy drugs, or tumor-feeding artery reselection were conducted for the second TACE procedure when an insufficient response after the first TACE occurred.

### Data Collection

Among 204 patients, 147 patients from hospital A and hospital B were assigned to a training set, and the remaining 57 patients from hospital C were assigned to a validation set (the training to validation ratio was approximately 7:3). The demographic, laboratory, and radiological data of patients were collected to assess the potential risk factors for early TACE refractoriness. The demographic and laboratory data included age, sex (male/female), Child-Pugh class (A/B), BCLC stage (0-A/B), underlying liver disease, history of resection, initial embolic agents (lipiodol/DEB), initial alpha-fetoprotein (AFP) level (≤400/>400 ug/L), and initial neutrophil to lymphocyte ratio (NLR). The radiological data included tumor distribution (unilobar/bilobar), number of tumors (solitary/2–3/>3), size of the largest tumor, and up-to-seven criteria (within/beyond). Patients who were beyond up-to-seven criteria was defined as: largest tumor diameter [cm] + number of tumors >7 (Mazzaferro et al., 2009; Koroki et al., 2020). Radiological data were independently reviewed by two radiologists with either 22 or 19 years of experience of abdominal imaging, respectively. Both of the radiologists were blinded to the clinical data and were not involved in the treatment. The final results of radiological data were made by the discussion between two radiologists.

### Follow-up Schedule and the Definition of TACE Refractoriness

Dynamic CT/MR imaging and laboratory variables were acquired before and after the first and the second TACE sessions. The treatment response of TACE was assessed by using dynamic CT/MR, and residual enhancement of nodules was measured with consideration of the 2019 version of Response Evaluation Criteria in *Cancer* of the Liver (RECICL) (Kudo et al., 2019).

The definition of TACE refractoriness was based on the JSH Consensus Guidelines as follows: 1) intrahepatic lesion: two or more consecutive ineffective responses was observed within the treated tumors (viable lesion>50%) or new lesion occurred in treated area, even after changing the chemotherapeutic agents or reanalysis of the feeding artery on response evaluation CT/MR after 1–3 months following adequate selective TACE; 2) AFP: continuous elevated levels of tumor markers right after TACE; 3) vascular invasion was observed; and 4) extrahepatic spread was observed.

The definition of early TACE refractoriness was that patients presented with TACE refractoriness after initial two consecutive TACE procedures.

### PFS Assessment

The PFS was defined as the time interval between date of TACE procedure and death whatever the cause, tumor progression or last clinical follow-up. Tumor progression was assessed according to the 2019 version of RECICL criteria (Kudo et al., 2019), which was defined as tumor enlargement of ≥50%, excluding the area of treatment-induced necrosis in either target lesion or non-target lesion. However, new intrahepatic lesion occurred in non-treated area after TACE was not defined as tumor progression.

### Statistical Analysis

The data were shown as the mean with standard deviation (SD), median with interquartile range (IQR), or frequency. To evaluate the inter-reader agreement of radiological data between the two abdominal radiologists, either intraclass correlation coefficient (ICC) analysis (for numerical data) or Kappa test (for categorical data) was performed. Agreement was classified as poor (ICC or Kappa value, 0–0.40), fair to good (ICC or Kappa value, 0.40–0.75), and excellent (ICC or Kappa value, >0.75). In univariate analysis, Pearson’s chi-squared test or Fisher’s exact test was used to compare categorical variables, while the independent sample t-test or rank-sum (Mann-Whitney) test was used to compare numerical variables. In multivariate analysis, a forward stepwise logistic regression model and nomogram were used. Variables with a *p*-value less than 0.05 in the univariate analysis were included in the multivariate model, and all those variables were tested by Diagnosis of Collinearity with variance inflation factors less than 5 (VIF < 5). The discrimination of this predictive model was examined by the receiver operating characteristic (ROC) curve, and the goodness of fit was validated by the Hosmer-Lemeshow test, in which a *p* value > 0.05 indicated good performance. Based on the factors selected by forward stepwise logistic regression, a one-to-one propensity score matching (PSM) was conducted to compare the PFS between patients who were present or absent of early TACE refractoriness. PFS curve was estimated by Kaplan-Meier method and compared by log-rank test.

Statistical analyses were performed with SPSS statistical software (SPSS version 20, International Business Machines Corporation) and R software (version 3.4.2, http://www.R-project.org). A probability value of <0.05 was considered statistically significant.

## Results

### Demographic and Laboratory Characteristics

Finally, a total of 204 patients were included (183 males and 21 females, with a mean age of 56.5 ± 11.6 years). All TACE procedures achieved technical success according to the Society of Interventional Radiology (SIR) guidelines (Gaba et al., 2016). The diagnosis of HCC was based on pathology (biopsy, n = 12) or on the American Association for the Study of Liver Practice Guidelines (n = 192). There were 181 (88.7%) patients with Child-Pugh class A and 23 patients with Child-Pugh class B (11.3%), 123 patients (60.3%) in BCLC stage 0-A and 81 patients (39.7%) in BCLC stage B. Patients with BCLC-0 or BCLC-A disease received DEB-TACE or conventional TACE for the following reasons: in cases beyond the Milan criteria, liver transplant was contraindicated; presence of portal hypertension or increased bilirubin, hepatectomy was contraindicated according to BCLC staging system; or for HCC lesions in unfavorable location, ablation was technically infeasible. Conventional chemoembolization was initially performed in 102 patients (102/204, 50.0%), and DEB-TACE was also initially performed in 102 patients (102/204, 50.0%). There were 127 patients with initial AFP ≤400 ug/L (62.3%) and 77 patients with AFP >400 ug/L (37.7%). The median of NLR was 2.95 (IQR 3.72).

### Radiological Characteristics

The inter-reader agreements of radiological data between the two radiologists were all excellent, with Kappa values of 0.947 (tumor distribution) and 0.954 (number of tumors), and the ICC value was 0.838 (size of the largest tumor). Among all patients, the sizes of the largest tumors were ≤50 mm in 83 patients (40.7%), 50–100 mm in 75 patients (36.8%), and >100 mm (22.5%) in 46 patients. Seventy-two (35.3%) patients had tumors with bilobar involvement, and 132 (64.7%) had tumors with unilobar involvement. One hundred twenty-three patients (60.3%) had a single tumor, 48 patients (23.5%) had two or three tumors, and 33 patients (16.2%) had more than three tumors. Eighty-one patients (39.7%) were within up-to-seven criteria, and 123 patients (60.3%) were beyond the up-to-seven criteria. The detailed demographic, radiological and laboratorial characteristics are summarized in Table 1.

**TABLE 1:** The demographic, radiological and laboratorial characteristics of the patients in training cohort and validation cohort.

Characteristics	Total (n = 204)	Training cohort (n = 147)	Validation cohort (n = 57)	*p* Value
Age (years)	56.5 ± 11.6	57.2 ± 12.5	54.7 ± 8.7	0.163
Gender (%)				0.656
Male	183 (89.7%)	131 (89.1%)	52 (91.2%)	
Female	21 (10.3%)	16 (10.9%)	5 (8.8%)	
Child-pugh class (%)				0.091
A	181 (88.7%)	127 (86.4%)	54 (94.7%)	
B	23 (11.3%)	20 (13.6%)	3 (5.3%)	
BCLC stage (%)				0.840
0-A	123 (60.3%)	88 (59.9%)	35 (61.4%)	
B	81 (39.7%)	59 (40.1%)	22 (38.6%)	
NLR	2.95 (IQR, 3.72)	2.39 (IQR, 1.73)	6.14 (IQR, 4.18)	<0.001
Underlying liver disease (%)				0.035
HBV	172 (84.3%)	119 (81.0%)	53 (92.9%)	
Other	10 (4.9%)	7 (4.8%)	3 (5.3%)	
None	22 (10.8%)	21 (14.2%)	1 (1.8%)	
Initial AFP (%)				0.037
≤400 ug/L	127 (62.3%)	98 (66.7%)	29 (50.9%)	
>400 ug/L	77 (37.7%)	49 (33.3%)	28 (49.1%)	
History of resection (%)				0.127
Presence	26 (12.7%)	22 (15.0%)	4 (7.0%)	
Absence	178 (87.3%)	125 (85.0%)	53 (93.0%)	
Tumor distribution (%)				0.539
Unilobar	132 (64.7%)	97 (66.0%)	35 (61.4%)	
Bilobar	72 (35.3%)	50 (34.0%)	22 (38.6%)	
Number of tumors (%)				0.601
Solitary	123 (60.3%)	88 (59.9%)	35 (61.4%)	
2–3	48 (23.5%)	33 (22.4%)	15 (26.3%)	
>3	33 (16.2%)	26 (17.7%)	7 (12.3%)	
Size of the largest tumor (%)				0.001
≤50 mm	83 (40.7%)	67 (45.6%)	16 (28.1%)	
50–100 mm	75 (36.8%)	57 (38.8%)	18 (31.6%)	
>100 mm	46 (22.5%)	23 (15.6%)	23 (40.3%)	
Up-to-seven criteria (%)				0.006
Within	81 (39.7%)	67 (45.6%)	14 (24.6%)	
Beyond	123 (60.3%)	80 (54.4%)	43 (75.4%)	
Initial embolic agents (%)				<0.001
Lipiodol	102 (50.0%)	60 (40.8%)	42 (73.7%)	
DEB	102 (50.0%)	87 (59.2%)	15 (26.3%)	

Note: HBV, hepatitis B virus; BCLC, Barcelona Clinic Liver *Cancer*; NLR, neutrophil to lymphocyte ratio; IQR, inter-quartile range; DEB drug-eluting beads.

### Potential Predictive Factors of Early TACE Refractoriness

**FIGURE 2:**
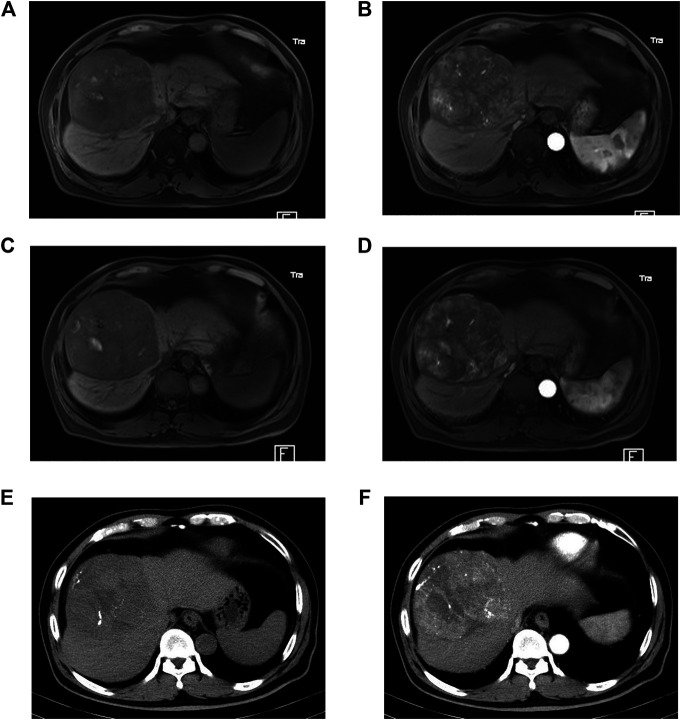
A 69 years old male with hepatocellular carcinoma (HCC) has undergone transarterial chemoembolization (TACE). Early TACE refractoriness is found after two consecutive TACE procedures. The baseline dynamic MR shows an 11 cm tumor with heterogeneous enhancement **(A, B)**. The first follow-up dynamic MR shows a viable tumor >50% **(C, D)**, and the second follow-up dynamic CT also shows a viable tumor >50% **(E, F)**.

The patterns of early TACE refractoriness in patients with HCC are illustrated in Table 2. Totally, there were 73 patients presented with early TACE refractoriness (35.8%, 73/204). A typical patient with early TACE refractoriness is shown in Figure 2. In univariate analysis, early TACE refractoriness was associated with BCLC stage (*p* < 0.001), tumor distribution (*p* < 0.001), number of tumors (*p* < 0.001), size of the largest tumor (*p* = 0.021), initial embolic agents (*p* = 0.045), and within/beyond up-to-seven criteria (*p* < 0.001). There was no statistical relationship between early TACE refractoriness and age, gender, Child-Pugh class, underlying liver disease, history of resection, initial AFP level, and NLR level. Multivariate analysis was performed using the significant risk factors determined in the univariate analysis, and within/beyond up-to-seven criteria (*p* = 0.001; odds ratio = 3.640, 95%CI 1.686–7.859), tumor distribution (*p* = 0.002; odds ratio = 3.251, 95%CI 1.536–6.883) and number of tumors (*p* = 0.005; odds ratio = 1.894, 95%CI 1.212–2.961) were independent predictive factors associated with early TACE refractoriness. The results from the univariable analysis performed on the training data set were summarized in Table 3.

**TABLE 2:** The patterns of early TACE refractoriness in patients with HCC.

Characteristics	Total (n = 204)	Training cohort (n = 147)	Validation cohort (n = 57)	*p* Value
Viable lesions >50%, n (%)	47 (23.0%)	28 (19.0%)	19 (33.3%)	0.093
Presence of new lesions, n (%)	7 (3.4%)	4 (2.7%)	3 (5.3%)	0.390
Vascular invasion, n (%)	9 (4.4%)	4 (2.7%)	5 (8.8%)	0.074
Extrahepatic spread, n (%)	5 (2.5%)	5 (3.4%)	0 (0.0)	-
Elevation of AFP, n (%)	31 (15.2%)	24 (16.3%)	7 (12.3%)	0.532

Note: TACE, transarterial chemoembolization; HCC, hepatocellularcarcinoma; AFP, alpha-fetoprotein.

**TABLE 3:** Assessment of potential risk factors of early TACE refractoriness in training cohort.

Characteristics	Absence of TACE refractoriness (n = 99)	Presence of TACE refractoriness (n = 48)	*p* Value	
			Univariate	Multivariate
Age (years)	57.6 ± 11.8	56.4 ± 14.0	0.588	-
Gender (%)			0.899	-
Male	88 (88.9%)	43 (89.6%)		
Female	11 (11.1%)	5 (10.4%)		
Child pugh class (%)			0.432	-
A	84 (84.8%)	43 (89.6%)		
B	15 (15.2%)	5 (10.4%)		
BCLC stage (%)			<0.001	-
0-A	70 (70.7%)	18 (37.5%)		
B	29 (29.3%)	30 (62.5%)		
NLR	2.39 (IQR, 1.84)	2.51 (IQR, 1.78)	0.687	
Underlying liver disease (%)			0.441	-
HBV	83 (83.8%)	36 (75.0%)		
Other	4 (4.0%)	3 (7.5%)		
None	12 (12.2%)	9 (22.5%)		
Initial AFP (%)			0.136	-
≤400 ug/L	70 (70.7%)	28 (58.3%)		
>400 ug/L	29 (29.3%)	20 (41.7%)		
History of resection (%)			0.687	-
Presence	14 (14.1%)	8 (16.6%)		
Absence	85 (85.9%)	40 (83.4%)		
Tumor distribution (%)			<0.001	0.002 (or, 3.251; 95%CI: 1.536–6.883)
Unilobar	79 (79.8%)	18 (37.5%)		
Bilobar	20 (20.2%)	30 (62.5%)		
Number of tumors (%)			<0.001	0.005 (or, 1.894; 95%CI: 1.212–2.961)
Solitary	70 (70.7%)	18 (37.5%)		
2–3	22 (22.2%)	11 (22.9%)		
>3	7 (7.1%)	19 (39.6%)		
Size of the largest tumor (%)			0.021	-
≤50 mm	53 (53.5%)	14 (29.2%)		
50–100 mm	33 (33.3%)	24 (50.0%)		
>100 mm	13 (13.2%)	10 (20.8%)		
Up-to-seven criteria (%)			<0.001	0.001 (or, 3.640; 95%CI: 1.686–7.859)
Within	56 (56.6%)	11 (22.9%)		
Beyond	43 (43.4%)	37 (77.1%)		
Initial embolic agent (%)			0.045	-
Lipiodol	46 (46.5%)	14 (29.2%)		
DEB	53 (53.5%)	34 (70.8%)		

Note: TACE, transarterial chemoembolization; NLR, neutrophil to lymphocyte ratio; IQR, interquartile range; HBV, hepatitis B virus; BCLC, Barcelona Clinic Liver *Cancer*; AFP, alpha-fetoprotein; DEB, drug-eluting beads.

### Predictive Model

**FIGURE 3:**
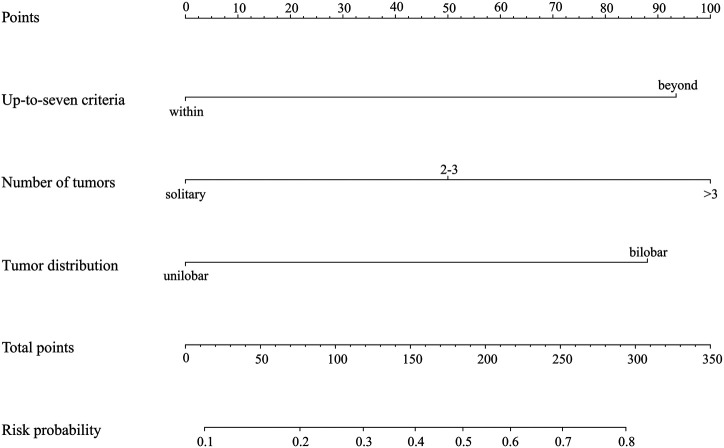
Nomogram to predict early TACE refractoriness, each predictor corresponds to a specific point by drawing a line straight upward to the points axis. Sum of the points is located on the total points axis, and the sum represents the probability of presenting early TACE refractoriness.

**FIGURE 4:**
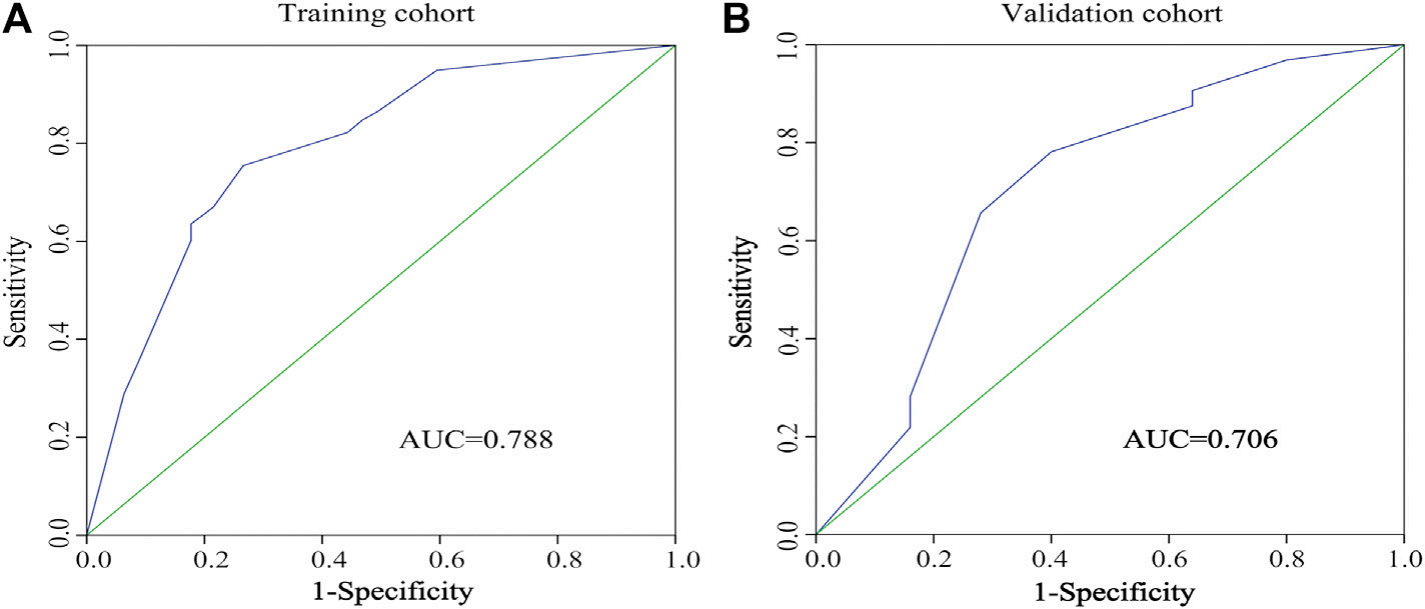
The receiver-operating characteristic (ROC) curve of the regression model in predicting early TACE refractoriness in training cohort **(A)** and validation cohort **(B)**.

A predictive model and nomogram (Figure 3) were built on the training set for predicting early TACE refractoriness based on within/beyond up-to-seven criteria, tumor distribution and number of tumors, with an area under the curve (AUC) of 0.788 (95%CI, 0.707–0.868), a sensitivity of 74.4% and a specificity of 73.8% (Figure 4A). While in the validation set, the AUC was 0.706 (95%CI, 0.564–0.848), with a sensitivity of 78.1% and a specificity of 60.0% (Figure 4B). Moreover, satisfactory calibration was confirmed by the Hosmer-Lemeshow test, with *p* values of 0.236 and 0.539 in the training and validation cohorts.

### Comparison of PFS

**FIGURE 5:**
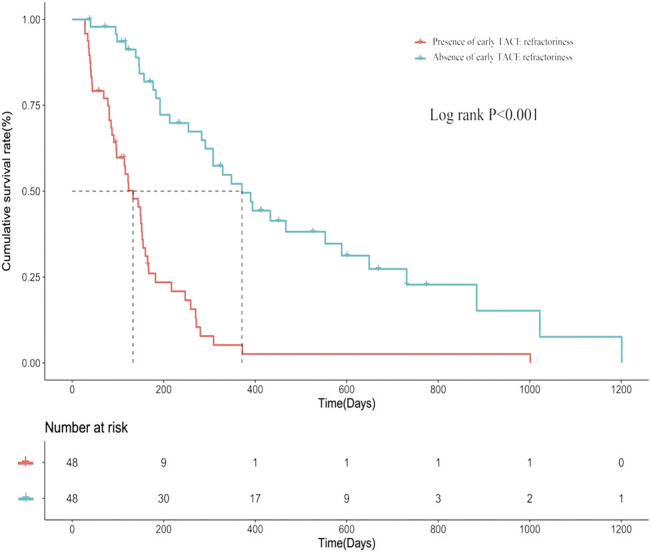
The progression-free survival (PFS) curves of patients who are present/absent of early TACE refractoriness.

Based on influencing factors selected by forward stepwise logistic regression including up-to-seven criteria, tumor distribution, and number of tumors, a PSM analysis was performed. After PSM, a total of 96 patients were enrolled, 48 of whom were present early TACE refractoriness, while 48 of whom were absent. There was no difference in baseline characteristics between two groups after PSM (Table 4). The median PFS in patients with or without early TACE refractoriness was 133 days (95% CI: 18.2–168.7) and 371 days (95% CI: 269.6–472.4), respectively. Patients who were absent of early TACE refractoriness had a significantly higher PFS rate than those of patients who were present (*p* < 0.001). The PFS curves of the two groups are shown in Figure 5.

**TABLE 4:** Demographic, radiological and laboratorial characteristics of the patients after propensity score matching.

Characteristics	Absence of TACE refractoriness (n = 48)	Presence of TACE refractoriness (n = 48)	*p* Value
Age (years)	56.1 ± 12.4	57.3 ± 12.5	0.625
Gender (%)			0.336
Male	41 (85.4%)	44 (91.7%)	
Female	7 (14.6%)	4 (8.3%)	
Child pugh class (%)			0.247
A	39 (81.3%)	43 (89.6%)	
B	9 (18.7%)	5 (10.4%)	
BCLC stage (%)			1.000
0-A	26 (54.2%)	26 (54.2%)	
B	22 (45.8%)	22 (45.8%)	
NLR	2.49 (IQR, 2.69)	2.65 (IQR, 3.58)	0.959
Underlying liver disease (%)			0.281
HBV	35 (72.9%)	41 (85.4%)	
Other	4 (8.3%)	3 (6.3%)	
None	9 (18.8%)	4(8.3%)	
Initial AFP (%)			0.294
≤400 ug/L	27 (56.3%)	32 (66.7%)	
>400 ug/L	21 (43.7%)	16 (33.3%)	
History of resection (%)			0.371
Presence	5 (10.4%)	8 (16.6%)	
Absence	43 (89.6%)	40 (83.4%)	
Tumor distribution (%)			1.000
Unilobar	27 (56.3%)	27 (56.3%)	
Bilobar	21 (43.7%)	21 (43.7%)	
Number of tumors (%)			1.000
Solitary	26 (54.2%)	26 (54.2%)	
2–3	16 (33.3%)	16 (33.3%)	
>3	6 (12.5%)	6 (12.5%)	
Size of the largest tumor (%)			0.638
≤50 mm	14 (29.2%)	18 (37.5%)	
50–100 mm	22 (45.8%)	18(37.5%)	
>100 mm	12 (25.0%)	12 (25.0%)	
Up-to-seven criteria (%)			0.824
Within	15 (31.3%)	14 (29.2%)	
Beyond	33 (68.7%)	34 (70.8%)	
Initial embolic agent (%)			0.063
Lipiodol	32 (66.7%)	23 (47.9%)	
DEB	16 (33.3%)	25 (52.1%)	

Note: TACE, transarterial chemoembolization; NLR, neutrophil to lymphocyte ratio; IQR, interquartile range; HBV, hepatitis B virus; BCLC, Barcelona Clinic Liver *Cancer*; AFP, alpha-fetoprotein; DEB, drug-eluting beads.

## Discussion

TACE is the standard and effective therapy for intermediate-stage HCC. However, this course of treatment can be limited in terms of effectiveness as patients present TACE refractoriness (Arizumi et al., 2017; Maesaka et al., 2020). For patients with TACE refractoriness, ineffective TACE should not be performed repeatedly, and those patients are recommended to switch to systemic therapy, such as sorafenib, as JSH suggested (Kudo et al., 2014). TACE refractoriness occurs almost inevitably, however, it should be noted that even in some patients, refractoriness presents in the very beginning of the TACE procedures (Maas et al., 2020).

In the present study, a predictive model was developed to predict the early TACE refractoriness, and this model was also validated in a validation cohort. The results showed that patients with the characteristics of tumor bilobar distribution, beyond up-to-seven criteria, and more than three tumors were significantly associated with early TACE refractoriness. The first predictor for early TACE refractoriness is beyond up-to-seven criteria. The up-to-seven criteria is one of a criteria for liver transplantation, while it is also used to predict the prognosis after TACE (Mazzaferro et al., 2009; Kimura et al., 2016). In Kimura’s study, they showed that the cumulative overall survival (OS) and disease-free survival (DFS) rates after TACE were higher in patients within up-to-seven criteria compared with those beyond the criteria (Kimura et al., 2016). More than three tumors is also an important predictor for early TACE refractoriness. It has been reported by Kim et al. that patients with the feature of multiple tumors (≥5) can significantly increase the risk of suffering TACE refractoriness, and the present study showed a similar finding (Kim et al., 2017). Multiple tumors was an indicator of the tumor burden and may represent the highly aggressive nature of the tumors, which predispose to the development of lesions at different sites. Prognosis of multiple tumors is worse compared to patients with solitary tumors, with five-year OS rates of 29.9% over 58%, respectively (Witjes et al., 2012; Dasari et al., 2020). Thus, patients with this feature may be more likely to present early TACE refractoriness. Another predictor is tumor distribution. To the best of our knowledge, this is the first study identifying bilobar involvement as a predictor for early TACE refractoriness. Bilobar involvement could be viewed as intrahepatic metastasis of the primary lesion, reflecting a more aggressive tumor behavior with a higher risk of consequent spread outside the liver (Elmoghazy et al., 2019; Dasari et al., 2020). The study from Elmoghazy et al. had revealed that HCC patients with bilobar involvement tended to have a higher probability of extrahepatic metastasis than those patients without bilobar involvement (Elmoghazy et al., 2019).

In predictive analysis, the regression model and nomogram showed moderate accuracy to predict early TACE refractoriness, with AUCs of 0.788 in the training set and 0.706 in the validation set, respectively. The clinical significance of this study is that it provides a relatively accurate, convenient, and noninvasive method for predicting early TACE refractoriness that is applicable to patients with HCC. Although TACE is recommended for unresectable HCC, not all patients can really benefit from TACE due to the heterogeneous population of unresectable HCC. Moreover, the present study performed a PSM analysis to compare the PFS rate between patients who were present or absent early TACE refractoriness, and the results showed that the PFS rates were significantly declined with patients who were present of early TACE refractoriness (*p* < 0.001), which indicated such patients might have a poor prognosis. Therefore, patients with high risk of early TACE refractoriness should be switched to systemic therapy as early as possible to improve prognosis and this study will certainly help distinguish patients with high risk of early TACE refractoriness.

Despite the valuable results described above, there are several limitations in the present study. Firstly, this is a retrospective study with a relatively small number of patients included and thus may be subject to selection and statistical bias. A prospective study with a relatively large study population should be performed to confirm this finding. Secondly, although those three variables above were tested by Diagnosis of Collinearity with variance inflation factors less than 5 (VIF < 5), there were still some confounding factors among the three variables. Thirdly, the OS of patients is not assessed, although OS is a crucial endpoint of prognosis for clinical study. Due to the retrospective nature of the present study, OS is quite difficult to obtain because some patients may not admit to hospital after tumor progression.

In conclusion, patients with characteristics of beyond up-to-seven criteria, bilobar tumor involvement, and more tumors are independent predictors of early TACE refractoriness, and patients with early TACE refractoriness may have a poor prognosis. Therefore, those characteristics should be taken into consideration when performing TACE.

## Data Availability

The raw data supporting the conclusions of this article will be made available by the authors, without undue reservation.
